# Supplementary material for: Coordinated multi-level adaptations across neocortical areas during task learning
Source: Nat Commun. 2025 Aug 19;16:7719. doi: 10.1038/s41467-025-62949-7 (PMC12365232; doi:10.1038/s41467-025-62949-7)
Supplement: Supplementary file 1 — Supplementary Information [file 41467_2025_62949_MOESM1_ESM.pdf]

Supplementary Information for:

Coordinated multi-level adaptations across neocortical areas during task learning

Shuting Han[1,2,3*], Fritjof Helmchen[1,2,3*].

[1]Brain Research Institute, University of Zurich, Zurich, Switzerland.

[2]Neuroscience Center Zurich (ZNZ), University of Zurich, Zurich, Switzerland.

[3]University Research Priority Program (URPP), Adaptive Brain Circuits in Development and Learning, University of Zurich, Zurich, Switzerland.

*Correspondence: han@hifo.uzh.ch (S.H); helmchen@hifo.uzh.ch (F.H)

This PDF file includes:

Supplementary Figures 1-11

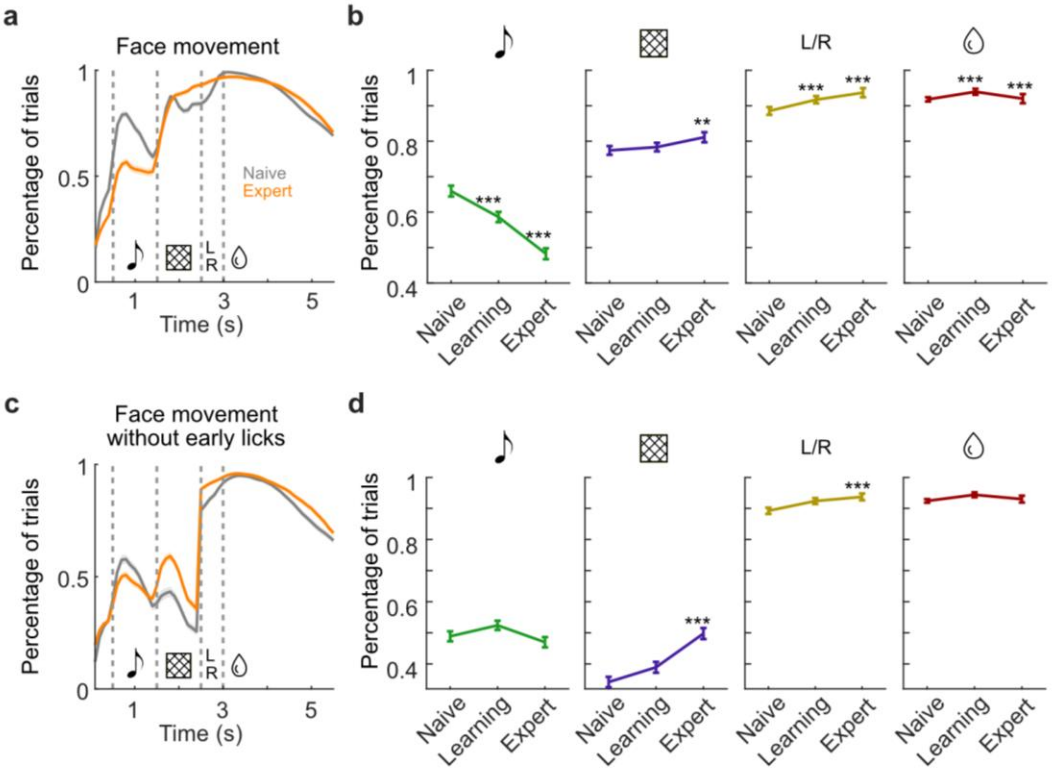

Supplementary Figure 1. Face movement with and without early licks

a. Percentage of trials with face movement during the task, in naive and expert mice. Face movement was defined as time points where the movement energy (frame-to-frame correlation of face image, normalized to [0, 1] for each session) was greater than 0.1. **b.** Quantification of (**a**) in separate task windows across learning stages. **c.** Percentage of trials with face movement during the task without early licks. In each trial, if there was an early lick (lick before choice window), all frames following the first early lick until choice onset were removed. **d.** Quantification of (**c**) in separate task windows across learning stages. Note that the face movement in tone window remain comparable across learning stages, indicating that the face movement in tone window was mostly due to early licks. Face movement in texture window increases during learning, indicating more whisking movement in expert phase. (15 mice, naive 134 sessions, learning 191 sessions, expert 166 sessions; **p<0.01, ***p<0.001; two-sided Wilcoxon rank-sum test against naive condition; mean ± SEM).

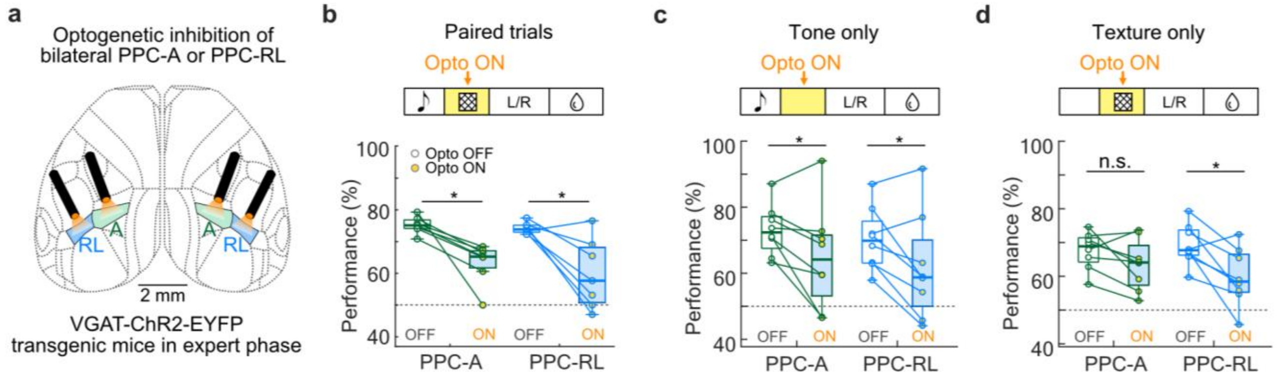

Supplementary Figure 2. PPC-A and PPC-RL are required for optimal task performance

a. Design of optogenetic inhibition experiment. Optical fibers were bilaterally implanted above PPC-A and PPC-RL of VGAT-ChR2 transgenic mice to allow optogenetic inhibition. **b.** Inhibition of either PPC-A or PPC-RL during the texture window reduced task performance. **c.** Inhibition of either PPC-A or PPC-RL during the texture window in tone-only condition reduced task performance. **d.** Inhibition of PPC-RL, but not PPC-A, during the texture window in texture-only condition reduced task performance. (Paired: 7 mice; tone only and texture only: 8 mice; *p<0.05; two-sided Wilcoxon signed rank paired test).

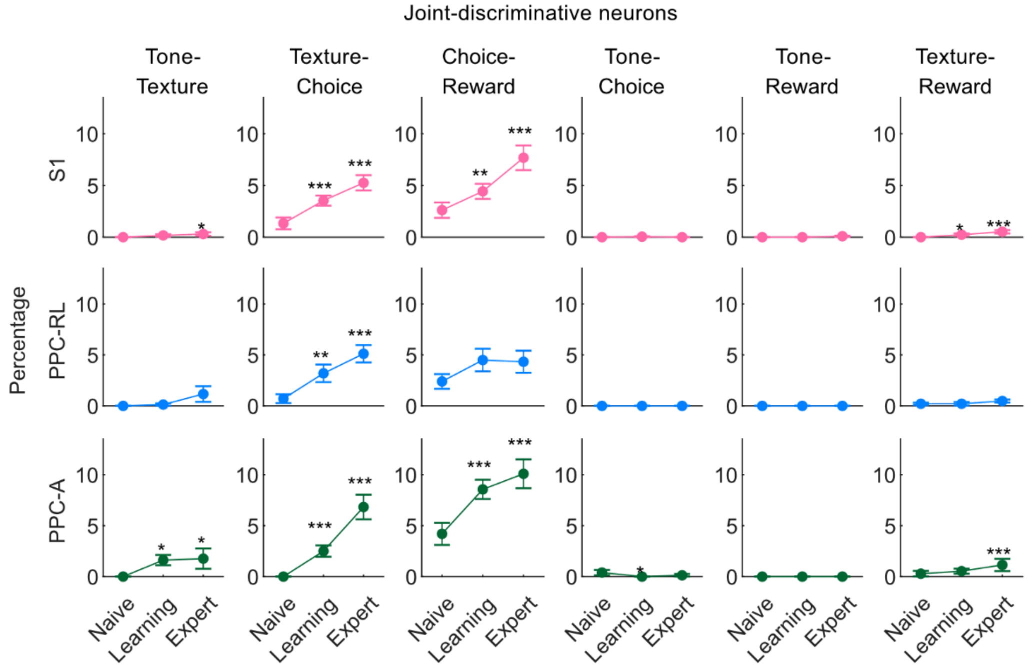

Supplementary Figure 3. Percentage of joint-discriminative neurons

Percentage of joint-discriminative neurons for pairs of task windows (labeled on top), for S1 (top row), PPC-RL (middle row), and PPC-A (bottom row). (S1: 13 mice, 130, 188, 149 sessions [naive, learning, expert]; PPC-RL: 13 mice, 70, 77, 104 sessions; PPC-A: 9 mice, 60, 111, 45 sessions; *$p<0.05$, **$p<0.01$, ***$p<0.001$; two-sided Wilcoxon rank-sum test against naive condition; mean ± SEM).

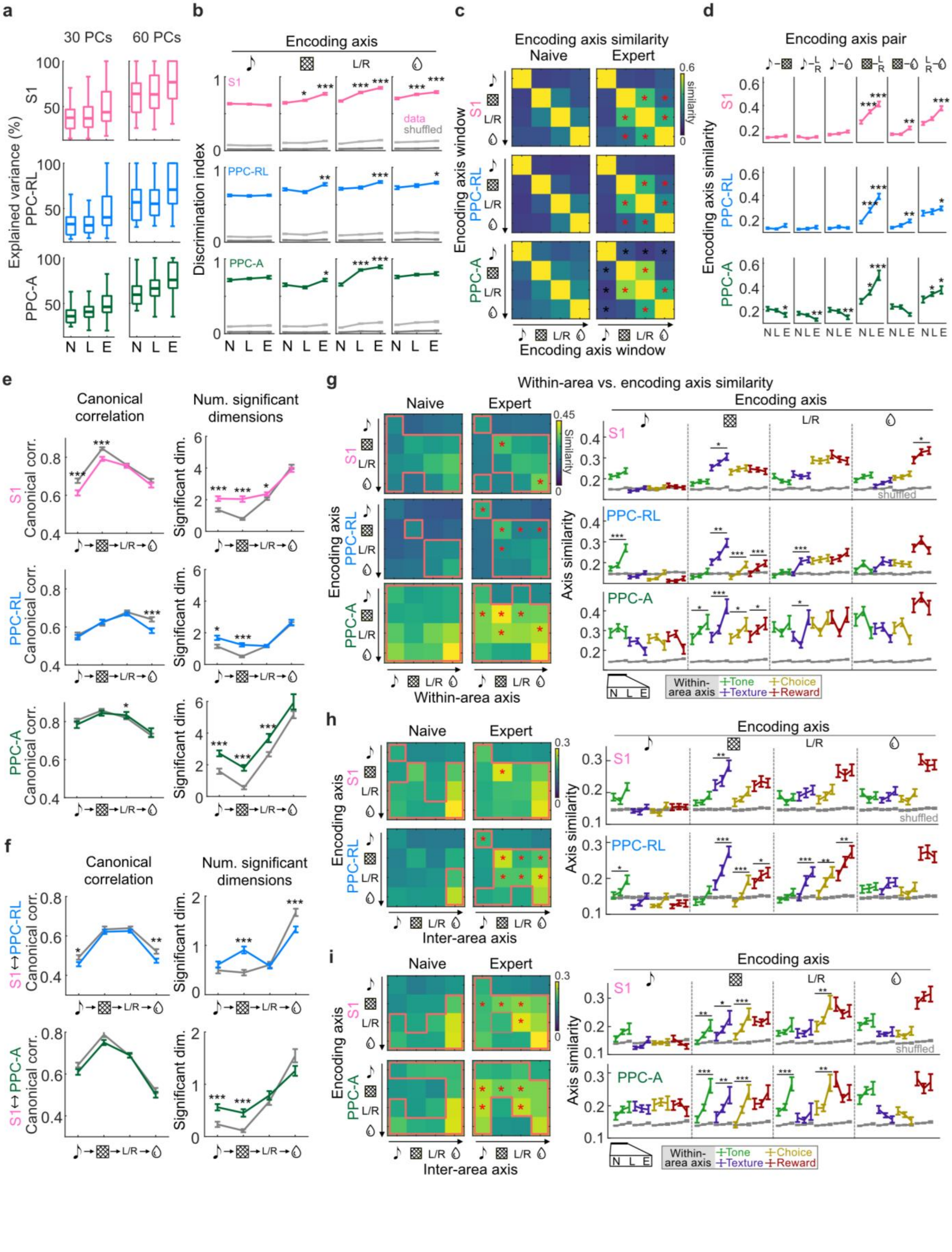

a
30 PCs
60 PCs
S1
PPC-RL
PPC-A
Explained variance (%)
N L E
b
Encoding axis
L/R
Discrimination index
data
shuffled
c
Encoding axis similarity
Naive
Expert
similarity
Encoding axis window
d
Encoding axis pair
Encoding axis similarity
e
Canonical correlation
Num. significant dimensions
Canonical corr.
Significant dim.
f
S1↔PPC-RL
S1↔PPC-A
g
Within-area vs. encoding axis similarity
Within-area axis
Axis similarity
shuffled
Within-area axis
Tone
Texture
Choice
Reward
h
Inter-area axis
i

Supplementary Figure 4. Subspace analysis using 60 PCs.

a. Explained variance by top 30 PCs (left) and 60 PCs (right) in S1 and PPC (N: naive; L: learning; E: expert). **b.** Discrimination index of projected population activity on the encoding axes using 60 PCs. **c.** Pairwise similarity of encoding axes for all task windows. Red and black asterisks indicate that the data from expert condition is significantly higher or lower than the naive condition. **d.** Quantification of pairwise encoding axes similarity. **e.** Canonical correlation strength (left panels) and number of significant dimensions (right panels) in naive and expert condition, in S1, PPC-RL, and PPC-A. **f.** Same as in (**e**) but for S1 and PPC-RL interaction (top) and S1 and PPC-A interaction (bottom). **g.** Left: pairwise similarity between the within-area interaction axis (x-axis) and the encoding axis (y-axis). Red boxes indicate that the data is above the shuffled distribution; red and black asterisks indicate that the data from expert condition is significantly higher or lower than the naive condition. Right: quantification of axis similarity. Line colors represent the within-area axis window; gray lines represent 95% quantile of shuffled data. **h.** Similarity between the inter-area axis and the encoding axis for S1 (top) and PPC-RL (bottom) interactions. Left: pairwise similarity between inter-area axis (x-axis) and encoding axis (y-axis) across task windows. Red boxes indicate that the data is above the corresponding shuffled distribution; red or black asterisks indicate that the data from expert condition is significantly higher or lower than the naive condition. Right: quantification of similarity. Line colors represent inter-area axis windows; gray lines represent 95% quantile of shuffled data. **i.** Same as in (**h**) but for S1 (top) and PPC-A (bottom) interactions. (S1: 13 mice, 122, 171, 119 sessions [naive, learning, expert]; PPC-RL: 13 mice, 66, 72, 86 sessions; PPC-A: 9 mice, 56, 99, 33 sessions; S1 and PPC-RL interaction: 13 mice, 66, 72, 86 sessions; S1 and PPC-A interaction: 9 mice, 56, 99, 33 sessions; *$p<0.05$, **$p<0.01$, ***$p<0.001$; (**c, g**, **h**, **i**) left panels: *$p<0.05$; two-sided Wilcoxon rank-sum test; mean ± SEM).

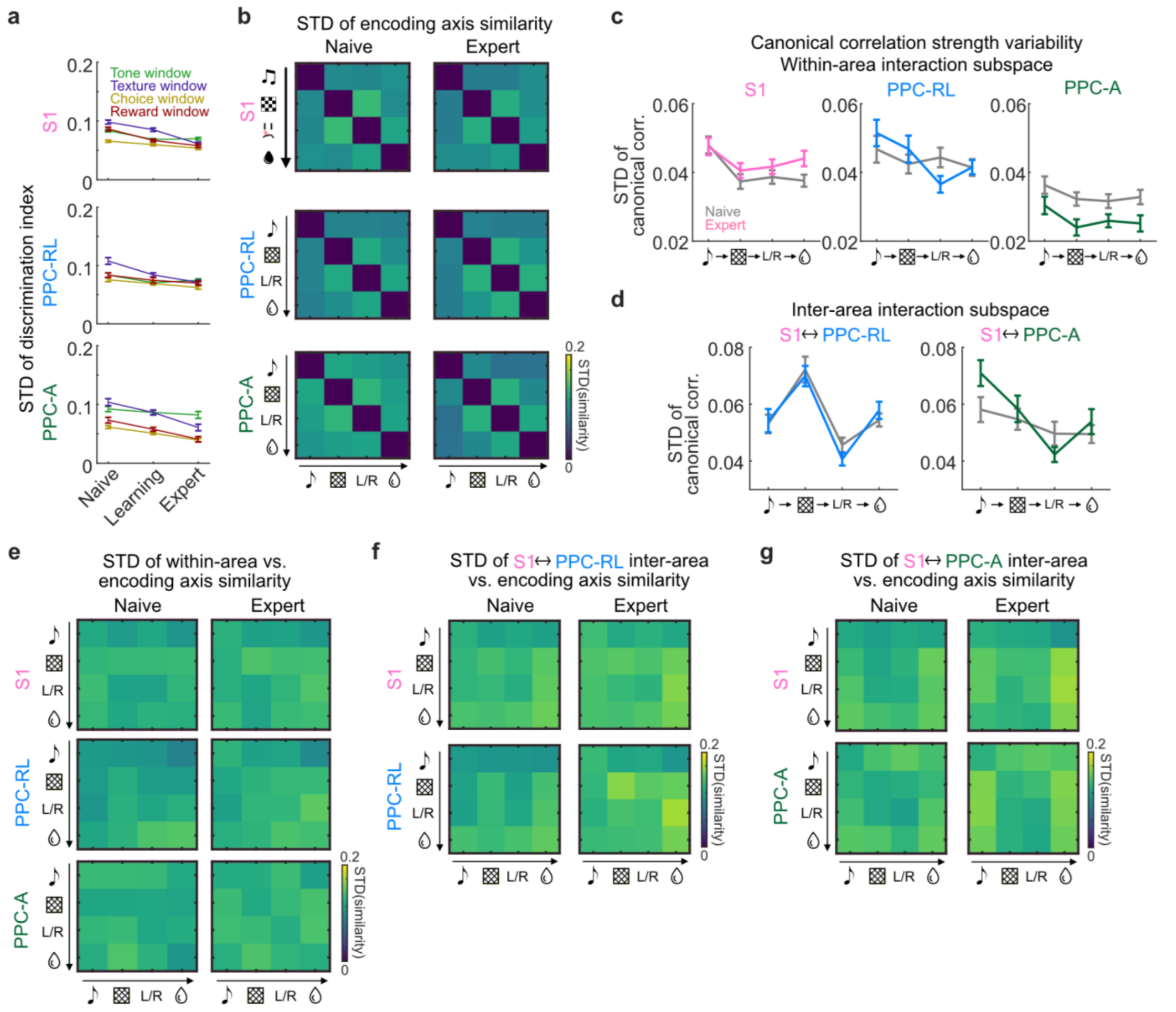

Supplementary Figure 5. Stability of subpopulation random splits

a. Standard deviation (STD) encoding axis discrimination index in each task window from Fig. 4c, using the 20 subpopulations from 10 random splits in each session. **b.** STD of encoding axis pairwise similarity of random splits. **c.** STD of CCA within-area correlation strength of random splits. **d.** STD of CCA inter-area correlation strength of random splits. **e.** STD of within-area axis similarity with encoding axis of random splits. **f.** STD of S1 and PPC-RL inter-area axis similarity with encoding axis of random splits. **g.** STD of S1 and PPC-A inter-area axis similarity with encoding axis of random splits. (S1: 13 mice, 122, 171, 119 sessions [naive, learning, expert]; PPC-RL: 13 mice, 66, 72, 86 sessions; PPC-A: 9 mice, 56, 99, 33 sessions; S1 and PPC-RL interaction: 13 mice, 66, 72, 86 sessions; S1 and PPC-A interaction: 9 mice, 56, 99, 33 sessions; mean ± SEM).

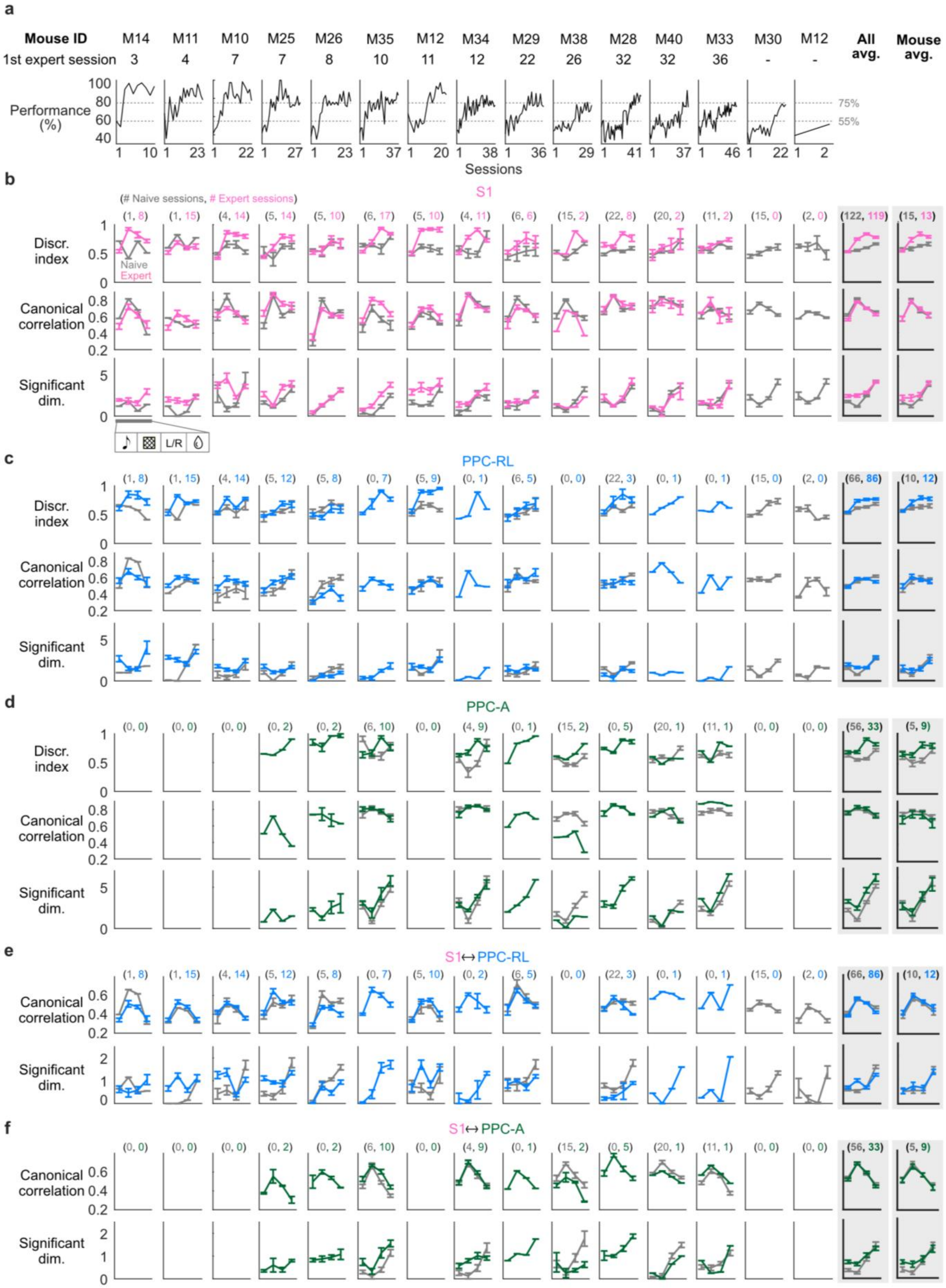
a
Mouse ID
1st expert session
Performance (%)
Sessions
b
S1
(# Naive sessions, # Expert sessions)
Discr. index
Canonical correlation
Significant dim.
Naive
Expert
All avg.
Mouse avg.
c
PPC-RL
d
PPC-A
e
S1↔PPC-RL
f
S1↔PPC-A

Supplementary Figure 6. Variability across individual mice

a. Learning curves of individual mice. The first session, in which each mouse's performance exceeded 75%, is labeled on top. Columns indicate individual mice; the second to last column (gray box) represents pooled results across mice and sessions; the last column (gray box) represents pooled results across mice, where sessions from the same mouse were averaged before pooling; this applies to all following panels. **b.** Discrimination index on the encoding axis (top), canonical correlation strength (middle) and number of significant dimensions (bottom) in naive and expert condition for each task window, in S1. Colored numbers in the brackets on top indicate the number of naive sessions (gray) and expert sessions (colored) for each mouse. The x-axis represents task windows. Mice showed variability that corresponded to the number of days to reach expert performance, and the total number of expert sessions. **c.** Same as in (**b**) for PPC-RL. **d.** Same as in (**b**) for PPC-A. **e.** Same as in (**b**) for S1 and PPC-RL interaction. **f.** Same as in (**b**) for S1 and PPC-A interaction. (S1: 13 mice, 122, 119 sessions [naive, expert]; PPC-RL: 13 mice, 66, 86 sessions; PPC-A: 9 mice, 56, 33 sessions; S1 and PPC-RL interaction: 13 mice, 66, 86 sessions; S1 and PPC-A interaction: 9 mice, 56, 33 sessions; mean ± SEM).

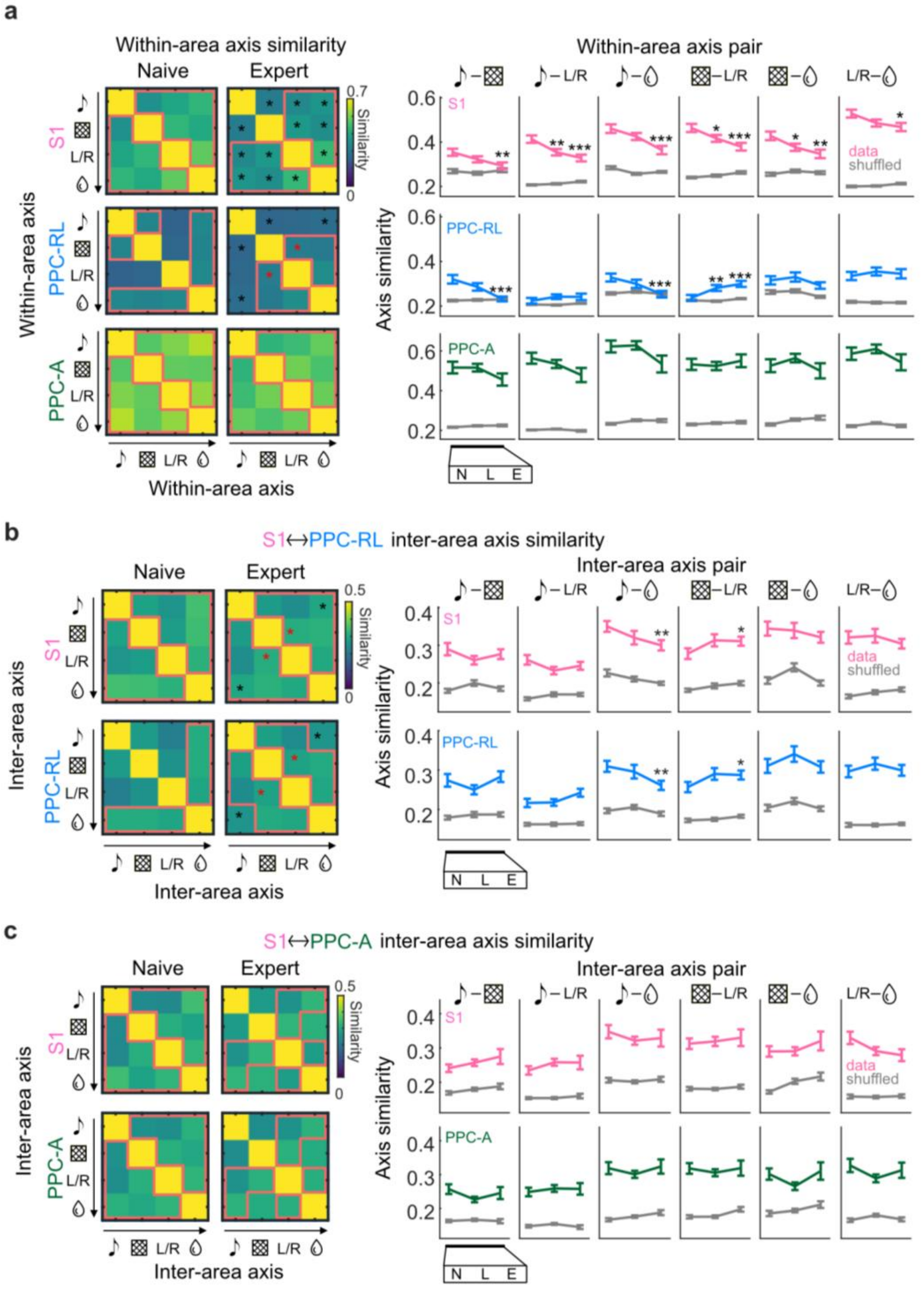

Supplementary Figure 7. Within-area axis similarity and inter-area axis similarity

a. Within-area axis pairwise similarity. Left: pairwise similarity of within-area axis across task windows. Red lines indicate that the data is above the corresponding shuffled distribution; asterisks indicate that the data from expert condition is significantly higher (red) or lower (black) than the naive condition. Right: quantification of similarity. **b.** Inter-area axis pairwise similarity for S1 (top) and PPC-RL (bottom). Left: pairwise similarity of inter-area axis across task windows. Right: quantification of similarity. **c.** Inter-area axis pairwise similarity for S1 (top) and PPC-A (bottom). Left: pairwise similarity of inter-area axis across task windows. Right: quantification of similarity. (S1: 13 mice, 122, 171, 119 sessions [naive, learning, expert]; PPC-RL: 13 mice, 66, 72, 86 sessions; PPC-A: 9 mice, 56, 99, 33 sessions; S1 and PPC-RL interaction: 13 mice, 66, 72, 86 sessions; S1 and PPC-A interaction: 9 mice, 56, 99, 33 sessions; left panels: *p<0.05; right panels: *p<0.05, **p<0.01, ***p<0.001; two-sided Wilcoxon rank-sum test against naive condition; mean ± SEM).

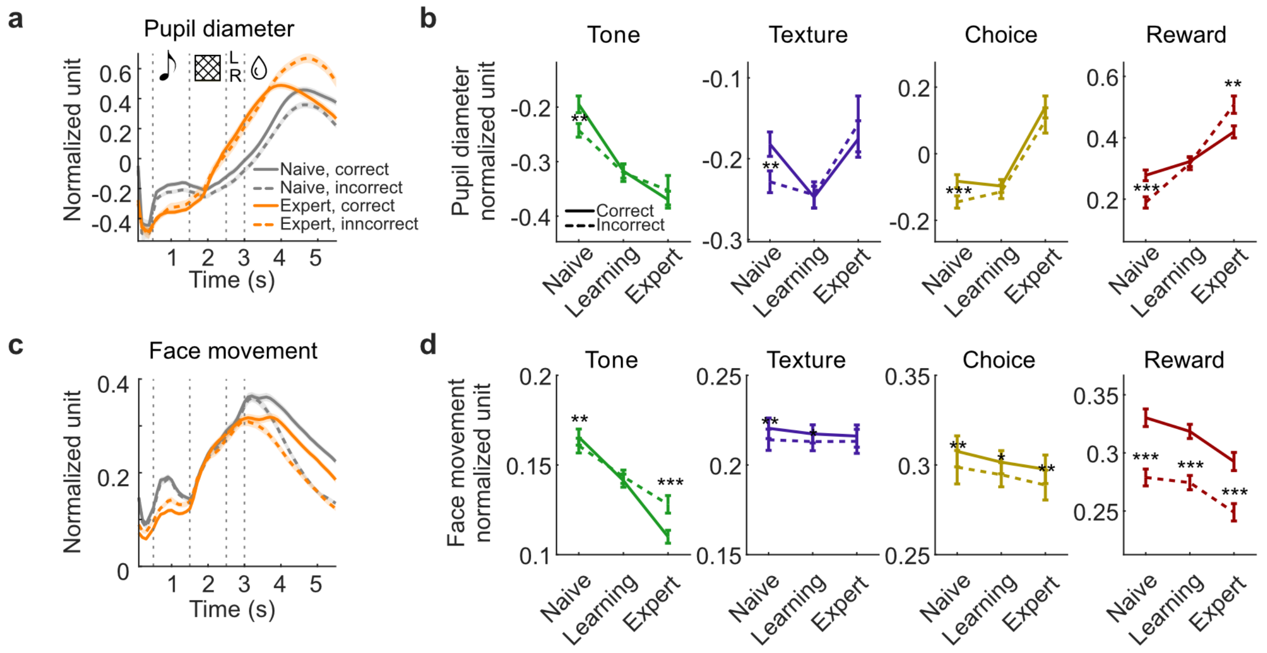

Supplementary Figure 8. Pupil diameter and face movement during incorrect trials

a. Normalized pupil diameter over the trial time, in correct and incorrect trials of naive and expert conditions. **b.** Quantification pupil diameter in correct and incorrect trials over learning. **c.** Normalized face movement over the trial time, in correct and incorrect trials of naive and expert conditions. **d.** Quantification face movement in correct and incorrect trials over learning. (15 mice, naive 134 sessions, learning 191 sessions, expert 166 sessions; **p*<0.05, ***p*<0.01, ****p*<0.001; two-sided Wilcoxon signed rank paired test between correct and incorrect trials; mean ± SEM).

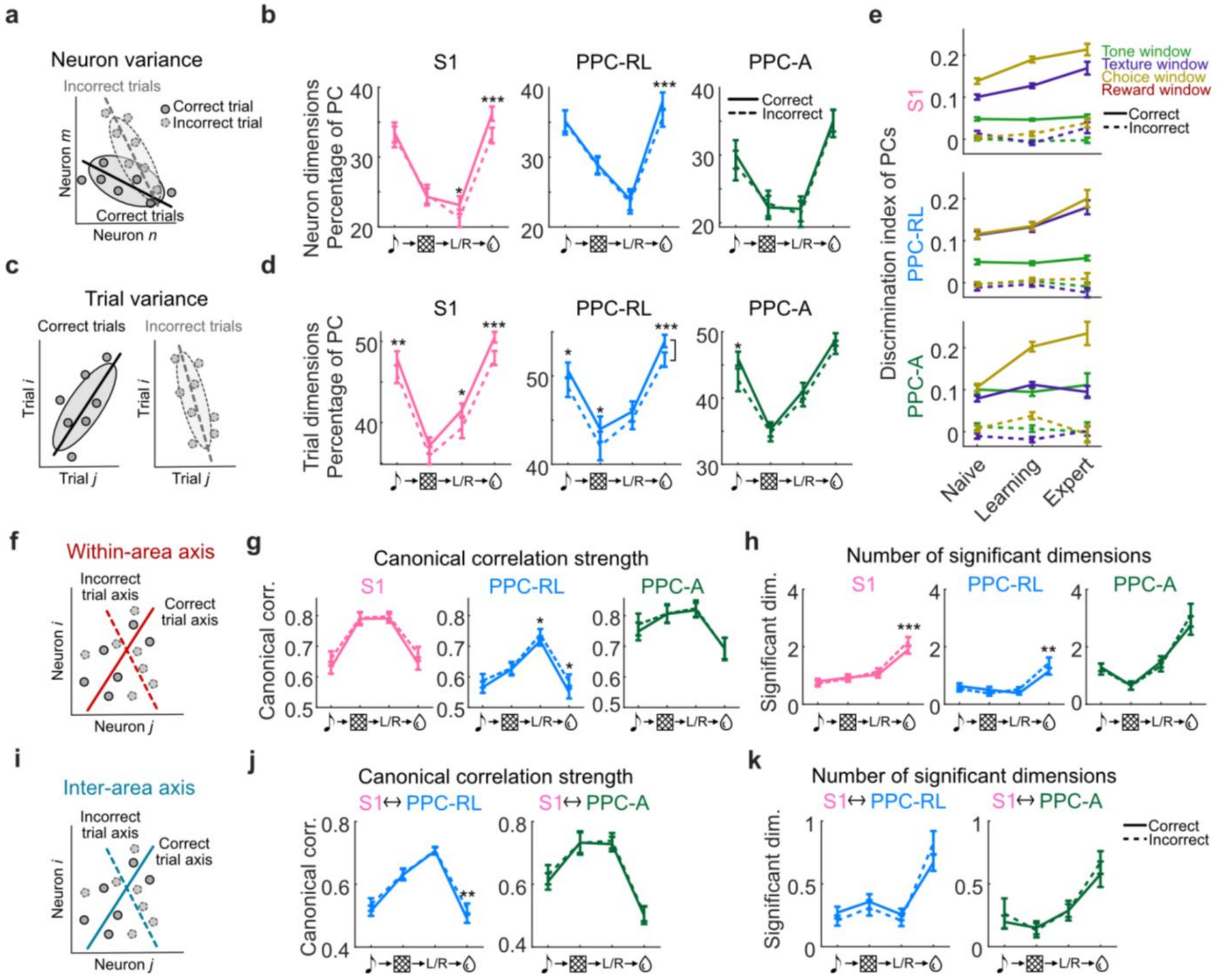

Supplementary Figure 9. Population dimensionality and interaction subspace in incorrect trials

a. Diagram of neuron variance axis in correct and incorrect trials. PCA was performed using correct or incorrect trials separately, with neurons as variables. **b.** Percentage of PCs that explains 70% of variance in neuron space during separate task windows, from correct trials (solid lines) and incorrect trials (dashed lines). **c.** Diagram of trial variance axis in correct and incorrect trials. PCA was performed using correct or incorrect trials separately, with trials as variables. **d.** Percentage of PCs that explains 70% of variance in trial space, over the trial time, from correct trials (solid lines) and incorrect trials (dashed lines). **e.** Session-averaged discrimination index of top 5 PCs, during correct trials (solid lines) and incorrect trials (dashed lines). **f.** Diagram of generating within-area axis from correct and incorrect trials. **g.** Inter-area canonical correlation strength for each task window in correct and incorrect trials. **h.** Number of significant dimensions of within-area interactions, in naive and expert condition for each task window in correct and incorrect trials. **i.** Diagram of generating inter-area axis from correct and incorrect trials. **j.** Within-area canonical correlation strength for each task window in correct (solid lines) and incorrect (dashed lines) trials. **k.** Number of significant dimensions of inter-area interactions for each task window in correct and incorrect trials. (S1: 13 mice, 149, 156, 48 sessions [naive, learning, expert]; PPC-RL: 13 mice, 79, 63, 33 sessions; PPC-A: 9 mice, 70, 93, 15 sessions; S1 and PPC-RL interaction: 13 mice, 79, 63, 33 sessions; S1 and PPC-A interaction: 9 mice, 70, 93, 15 sessions; *p<0.05, **p<0.01, ***p<0.001; two-sided Wilcoxon signed rank paired test between correct and incorrect trials; mean ± SEM).

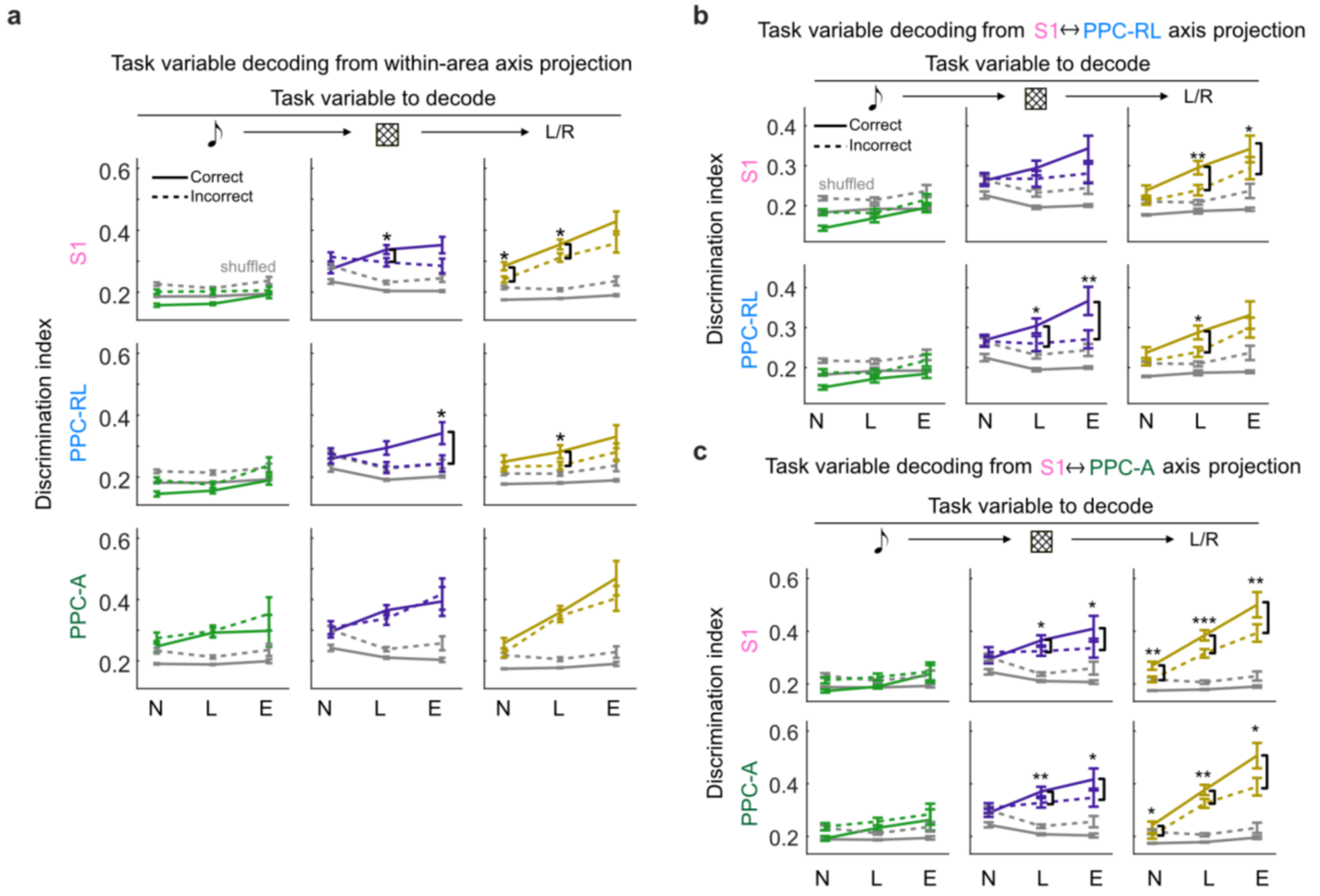

Supplementary Figure 10. Discrimination index of within- and inter-area activity in incorrect trials

a. The absolute value of discrimination index of population activity projected onto the within-area axes for S1 (top row), PPC-RL (middle), and PPC-A (bottom). Decoding was performed on the projections from the population activity during matching task windows and axes (e.g., decoding texture using texture window activity projected onto the within-area axis of texture window). Solid lines represent correct trials, dashed lines represent incorrect trials; gray lines represent 95% quantile of shuffled data. **b.** The absolute value of discrimination index of population activity projected onto the S1 and PPC-RL inter-area axes. **c.** The absolute value of discrimination index of population activity projected onto the S1 and PPC-A inter-area axes. (S1: 13 mice, 149, 156, 48 sessions [naive, learning, expert]; PPC-RL: 13 mice, 79, 63, 33 sessions; PPC-A: 9 mice, 70, 93, 15 sessions; S1 and PPC-RL interaction: 13 mice, 79, 63, 33 sessions; S1 and PPC-A interaction: 9 mice, 70, 93, 15 sessions; **p*<0.05, ***p*<0.01, ****p*<0.001; two-sided Wilcoxon signed rank paired test between correct and incorrect trials; mean ± SEM).

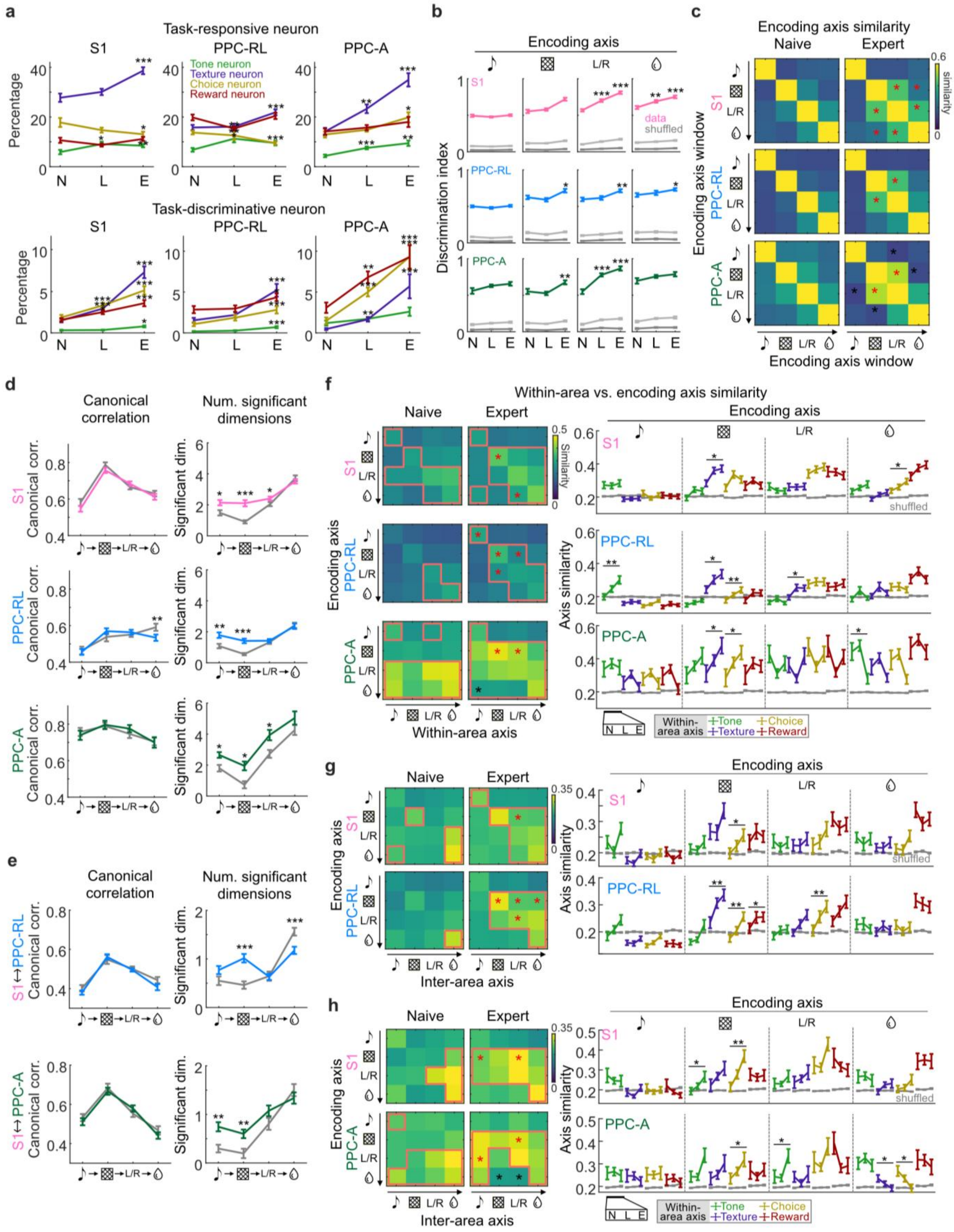

a
Task-responsive neuron
S1
PPC-RL
PPC-A
Percentage
Tone neuron
Texture neuron
Choice neuron
Reward neuron
Task-discriminative neuron
b
Encoding axis
Discrimination index
data
shuffled
c
Encoding axis similarity
Naive
Expert
similarity
Encoding axis window
d
Canonical correlation
Num. significant dimensions
Canonical corr.
Significant dim.
e
f
Within-area vs. encoding axis similarity
Similarity
Within-area axis
Axis similarity
Within-area axis
g
Inter-area axis
h

Supplementary Figure 11. Analysis results using one session per day

a. Percentage of task-responsive neurons (top) and task-discriminative neurons (bottom) in S1, PPC-RL, and PPC-A. Colors represent the task window to which the neurons are responsive. Only one session per day was used for pooled statistics, in order to avoid correlated results from multiple sessions from training days with high number of trials. The session with the highest performance was taken from each day. **b.** Discrimination index of population activity projection on the encoding axes, for each task variable, in S1, PPC-RL, and PPC-A. **c.** Pairwise similarity of encoding axes. Red and black asterisks indicate that the data from expert condition is significantly higher or lower than the naive condition. **d.** Canonical correlation strength (left) and number of significant dimensions (right) in naive and expert condition for each task window, in S1, PPC-RL, and PPC-A. **e.** Canonical correlation strength (left) and number of significant dimensions (right) for S1 and PPC-RL interaction (top) and S1 and PPC-A interaction (bottom). **f.** Left: pairwise similarity between the within-area interaction axis (x-axis) and the encoding axis (y-axis) across task windows. Red boxes indicate that the data is above the shuffled distribution; red and black asterisks indicate that the data from expert condition is significantly higher or lower than the naive condition. Right: quantification of axis similarity. Line colors represent the within-area axis window; gray lines represent 95% quantile of shuffled data. **g.** Inter-area axis similarity with encoding axis in S1 (top) and PPC-RL (bottom). Left: pairwise similarity between inter-area axis (x-axis) and encoding axis (y-axis). Right: quantification of similarity. **h.** Same plots as in (**g**) for S1 (top) and PPC-A (bottom) interaction. (S1: 13 mice, 51, 89, 75 sessions [naive, learning, expert]; PPC-RL: 13 mice, 34, 38, 52 sessions; PPC-A: 9 mice, 17, 51, 23 sessions; S1 and PPC-RL interaction: 13 mice, 34, 38, 52 sessions; S1 and PPC-A interaction: 9 mice, 17, 51, 23 sessions; *p<0.05, **p<0.01, ***p<0.001; (**c**, **f**, **g**, **h**) left panels: *p<0.05; two-sided Wilcoxon rank-sum test against naive condition; mean ± SEM).
