# Supplementary material for: Coordinated multi-level adaptations across neocortical areas during task learning
Source: Nat Commun. 2025 Aug 19;16:7719. doi: 10.1038/s41467-025-62949-7 (PMC12365232; doi:10.1038/s41467-025-62949-7)
Supplement: Supplementary file 3 — Reporting Summary [file 41467_2025_62949_MOESM3_ESM.pdf]

nature portfolio

Corresponding author(s): Shuting Han, Fritjof Helmchen

Last updated by author(s): Jul 1, 2025

Reporting Summary

Nature Portfolio wishes to improve the reproducibility of the work that we publish. This form provides structure for consistency and transparency in reporting. For further information on Nature Portfolio policies, see our Editorial Policies and the Editorial Policy Checklist.

Statistics

For all statistical analyses, confirm that the following items are present in the figure legend, table legend, main text, or Methods section.

n/a	Confirmed	
☐	☒	The exact sample size (*n*) for each experimental group/condition, given as a discrete number and unit of measurement
☐	☒	A statement on whether measurements were taken from distinct samples or whether the same sample was measured repeatedly
☐	☒	The statistical test(s) used AND whether they are one- or two-sided<br>*Only common tests should be described solely by name; describe more complex techniques in the Methods section.*
☒	☐	A description of all covariates tested
☐	☒	A description of any assumptions or corrections, such as tests of normality and adjustment for multiple comparisons
☐	☒	A full description of the statistical parameters including central tendency (e.g. means) or other basic estimates (e.g. regression coefficient) AND variation (e.g. standard deviation) or associated estimates of uncertainty (e.g. confidence intervals)
☐	☒	For null hypothesis testing, the test statistic (e.g. *F*, *t*, *r*) with confidence intervals, effect sizes, degrees of freedom and *P* value noted<br>*Give P values as exact values whenever suitable.*
☒	☐	For Bayesian analysis, information on the choice of priors and Markov chain Monte Carlo settings
☒	☐	For hierarchical and complex designs, identification of the appropriate level for tests and full reporting of outcomes
☒	☐	Estimates of effect sizes (e.g. Cohen's *d*, Pearson's *r*), indicating how they were calculated

Our web collection on statistics for biologists contains articles on many of the points above.

Software and code

Policy information about availability of computer code

Data collection	Behavior training: custom LabVIEW software (HIFOTDT 1.2.7)<br>Body camera: custom LabVIEW software<br>Widefield imaging: custom LabVIEW software<br>Multiarea two photon imaging: custom C++ software (Scope, http://rkscope.sourceforge.net)
Data analysis	Two photon data processing: open source python pipeline Suite2p (https://github.com/MouseLand/suite2p)<br>Canonical correlation analysis: open source MATLAB package (https://github.com/joao-semedo/canonical-correlation-maps)<br>All other analysis was performed with custom written MATLAB code using MATLAB R2018b and R2024b (https://github.com/HelmchenLabSoftware/multiarea_learning_manuscript).

For manuscripts utilizing custom algorithms or software that are central to the research but not yet described in published literature, software must be made available to editors and reviewers. We strongly encourage code deposition in a community repository (e.g. GitHub). See the Nature Portfolio guidelines for submitting code & software for further information.

Data

Policy information about availability of data

All manuscripts must include a data availability statement. This statement should provide the following information, where applicable:

- Accession codes, unique identifiers, or web links for publicly available datasets
- A description of any restrictions on data availability
- For clinical datasets or third party data, please ensure that the statement adheres to our policy

The data that support the findings of this study are available from the corresponding author upon request.

Research involving human participants, their data, or biological material

Policy information about studies with human participants or human data. See also policy information about sex, gender (identity/presentation), and sexual orientation and race, ethnicity and racism.

Reporting on sex and gender	No human subjects participated in this study.
Reporting on race, ethnicity, or other socially relevant groupings	No human subjects participated in this study.
Population characteristics	No human subjects participated in this study.
Recruitment	No human subjects participated in this study.
Ethics oversight	No human subjects participated in this study.

Note that full information on the approval of the study protocol must also be provided in the manuscript.

Field-specific reporting

Please select the one below that is the best fit for your research. If you are not sure, read the appropriate sections before making your selection.

☒ Life sciences ☐ Behavioural & social sciences ☐ Ecological, evolutionary & environmental sciences

For a reference copy of the document with all sections, see nature.com/documents/nr-reporting-summary-flat.pdf

Life sciences study design

All studies must disclose on these points even when the disclosure is negative.

Sample size	No statistical methods were used to pre-determine sample sizes, but our sample sizes are similar to those reported in previous publications. In behavioral experiments where neuronal populations were recorded, previous literature typically report 5-15 mice per experiment group, and >3 sessions per mouse. Our sample size was in accordance with these practice.
Data exclusions	No data was excluded from the study.
Replication	All analysis were performed across multiple mice, and multiple sessions for each mouse and each imaging condition. The results were reproduced, as indicated by the statistical tests. Bootstrapping and random re-sampling were applied whenever possible (Fig. 4-7), and the analysis results were reproduced with multiple models generated on separate subsets of data within session.
Randomization	Group assignment was inclusive. Out of all mice that were used for two-photon imaging, 9 mice were included in both experiment groups (S1-PPCA and S1-PPCRL imaging sessions, as well as single modality experiments), and 5 additional mice were included only in S1-PPCRL imaging sessions.
Blinding	No blinding was done in this study. Data of different experiment designs was collected on the same mice, therefore blinding during data collection was not applicable. The analysis pipeline was developed according to the biological questions we wanted to address, and applied to each group separately afterwards.

Reporting for specific materials, systems and methods

We require information from authors about some types of materials, experimental systems and methods used in many studies. Here, indicate whether each material, system or method listed is relevant to your study. If you are not sure if a list item applies to your research, read the appropriate section before selecting a response.

Materials & experimental systems

n/a	Involved in the study
☒	☐ Antibodies
☒	☐ Eukaryotic cell lines
☒	☐ Palaeontology and archaeology
☐	☒ Animals and other organisms
☒	☐ Clinical data
☒	☐ Dual use research of concern
☒	☐ Plants

Methods

n/a	Involved in the study
☒	☐ ChIP-seq
☒	☐ Flow cytometry
☒	☐ MRI-based neuroimaging

Animals and other research organisms

Policy information about studies involving animals; ARRIVE guidelines recommended for reporting animal research, and Sex and Gender in Research

Laboratory animals	The detailed animal information is reported in the Methods section. Strains used in this study: (1) RasGRF2a-dCre;CamK2a-tTA;TITL-GCaMP6f; (2) GP5.17; (3) Snap25-IRES2-Cre-D;CamK2a-tTA;TITL-GCaMP6f; (4) RasGRF2a-dCre;tTA2-GCaMP6f. Both sexes were used. Mice were 2.5-4 months old at the beginning of behavior training, and 3-5 months old at the time of experiment. Mice were housed on a 12-h reversed light/dark cycle at an ambient temperature of between 21 °C and 23 °C and humidity between 55% and 60%.
Wild animals	No wild animals were used.
Reporting on sex	Both sexes were used (9 males, 8 females) and randomly assigned to each group. The analysis was done using data from both sexes together. No sex-based analysis was performed, since sex was not a variable of interest in this study.
Field-collected samples	This study did not involve samples collected from the field.
Ethics oversight	All experimental procedures were carried out in accordance with the guidelines of the Federal Veterinary Office of Switzerland and were approved by the Cantonal Veterinary Office in Zurich.

Note that full information on the approval of the study protocol must also be provided in the manuscript.

Plants

Seed stocks	No plants were used in this study.
Novel plant genotypes	No plants were used in this study.
Authentication	No plants were used in this study.
